# Closing the DNA replication cycle: from simple circular molecules to supercoiled and knotted DNA catenanes

**DOI:** 10.1093/nar/gkz586

**Published:** 2019-07-05

**Authors:** Jorge B Schvartzman, Pablo Hernández, Dora B Krimer, Julien Dorier, Andrzej Stasiak

**Affiliations:** 1 Department of Cell and Molecular Biology, Centro de Investigaciones Biológicas (CSIC), Ramiro de Maeztu 9, 28040 Madrid, Spain; 2 SIB Swiss Institute of Bioinformatics, 1015 Lausanne, Switzerland; 3 Center for Integrative Genomics, Faculty of Biology and Medicine, University of Lausanne, 1015 Lausanne, Switzerland

## Abstract

Due to helical structure of DNA, massive amounts of positive supercoils are constantly introduced ahead of each replication fork. Positive supercoiling inhibits progression of replication forks but various mechanisms evolved that permit very efficient relaxation of that positive supercoiling. Some of these mechanisms lead to interesting topological situations where DNA supercoiling, catenation and knotting coexist and influence each other in DNA molecules being replicated. Here, we first review fundamental aspects of DNA supercoiling, catenation and knotting when these qualitatively different topological states do not coexist in the same circular DNA but also when they are present at the same time in replicating DNA molecules. We also review differences between eukaryotic and prokaryotic cellular strategies that permit relaxation of positive supercoiling arising ahead of the replication forks. We end our review by discussing very recent studies giving a long-sought answer to the question of how slow DNA topoisomerases capable of relaxing just a few positive supercoils per second can counteract the introduction of hundreds of positive supercoils per second ahead of advancing replication forks.

## INTRODUCTION

Watson and Crick’s ingenious inference that DNA forms a double-stranded helix with about 10 base pair per turn has immediately posed the questions of how DNA strands are separated during DNA replication without encountering topological difficulties (1). At that very early time of molecular biology, not only DNA polymerases but also DNA topoisomerases were not yet discovered but despite this Watson and Crick prophesized that ‘Although it is difficult at the moment to see how these processes occur without everything getting tangled, we do not feel that this objection will be insuperable’ (1). Today, 66 years after the prophecy of Watson and Crick, we are getting a more complete picture of how the action of DNA topoisomerases makes it possible that DNA replication proceeds without everything getting tangled. This picture shows that despite the presence of several kinds of specialized topoisomerases that act on replicating DNA molecules, there is quite a lot of various forms of DNA tangling implicated in the process of DNA replication. There is DNA supercoiling, DNA catenation and DNA knotting and their various combinations. We discuss here different forms of DNA tangling observed during DNA replication and also discuss why some forms of that tangling are beneficial to the process of DNA replication.

### Effect of torsional stress on the shape and properties of DNA molecules

DNA molecules are thin, springy filaments that oppose bending and torsional deformations (2). Reacting to torsional stress that is generated by various biological mechanisms, DNA molecules minimize their elastic energy by forming superhelical structures. Importantly, the mechanical resistance to torsional/twisting deformations requires the continuity of both strands of the DNA duplex. In supercoiled circular DNA molecules, it suffices that one strand of the duplex is nicked to dissipate all torsional stress by swiveling the free ends of the cut strand around the intact strand at the site of the nick. To understand how torsional stress contributes to the panoply of shapes taken by DNA molecules undergoing various biological transactions, such as DNA replication, it is very helpful to become familiar with DNA topology.

### Primary and more advanced concepts in DNA topology

**Figure 1.**
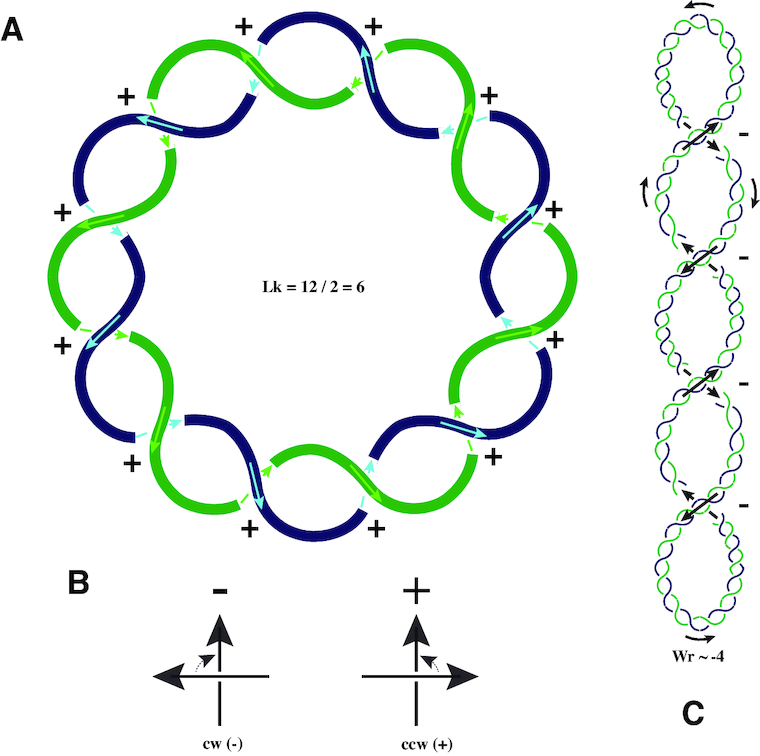
Crossing sign convention, linking number (Lk) and writhe (Wr). (**A**) Schematic presentation of a double-stranded DNA minicircle. For topological considerations both strands of DNA are given the same direction along the circle. Therefore, for right-handed DNA helix the intra-duplex inter-strand crossings have positive signs. The linking number (Lk) of the presented DNA minicircle is 7 (see the main text). The twist (Tw) of that minicircle is also 7 as the helix makes 7 complete right-handed turns. (**B**) Crossings between two oriented segments can be only of positive or negative sign. Crossing has negative sign if the direction arrow that is closer to the observer would need to be turned in a clockwise direction to overly it with the direction arrow that is further from the observer. If that direction of turning is counter-clockwise, the crossing has positive sign. Of course, the turning angle cannot be larger than 180°. (**C**) In negatively supercoiled DNA molecule forming a regular superhelix, the self-crossings of DNA axis have negative signs. Notice that orientation of the underlying and overlying direction arrows at each crossing are not independent from each other but result from assigning a consistent direction (see the black arrows) along the whole DNA molecule analyzed. The DNA double-helix is shown in blue and green.

One important descriptor of the DNA topology of covalently closed circular molecules is the linking number (Lk) that tells us how many times and with what handedness the two strands forming the double-helix of a given DNA molecule are linked with each other. The absolute value of the DNA Lk corresponds to the minimal number of passages of one strand through the other needed to completely separate the two strands (3). Such inter-strand passages that can be catalyzed by type I topoisomerases (see later) consist of transient cutting of one strand, passing the other strand through the cut and then resealing the cut strand. By convention, for topological considerations needed to define DNA Lk, the same direction is given to both strands of the DNA double-helix despite the opposing 5′-3′ directionality of the two strands (2) (see Figure 1A). This is why B-DNA that forms a right-handed helix (4) has a positive sign for Lk whereas a left-handed helix, such as Z-DNA (5), would have a negative Lk when closed into a covalently closed circle. Assuming that we have high resolution images of DNA molecules permitting us to trace individual strands (as schematically shown in Figure 1A), and that we want to determine the Lk of such DNA molecules, we need first to arbitrarily assign directions to both strands but remembering that for topological considerations of DNA structure both strands need to run in the same direction along the helix. Then using the convention of positive and negative crossings (Figure 1B), we can score all the crossings of one curve with the other in a given projection and add the contributions of every crossing. Since each complete encircling of one strand around the other introduces two crossings, each positive and each negative crossing counts as +1/2 or −1/2, respectively. Importantly, the sum of crossing scoring in a given projection always gives an integer value and for a given pair of closed curves in space this value is the same in every direction of viewing. In addition, the Lk of two closed curves in space remains the same upon any continuous deformation such a stretching, twisting or bending, which can be verified by crossing scoring before and after any continuous deformation. In other words, Lk is a topological invariant (2). An additional important property of DNA Lk is that it is always equal to the sum of two geometrical variables of a given covalently closed DNA molecule: Twist (Tw) and Writhe (Wr) according to the equation: Lk = Tw + Wr, where Tw is the number of helical turns of the DNA double-helix, which corresponds to the integrated Tw angle (expressed in the number of 360° rotations) between all successive base pairs in the entire DNA molecule.

**Figure 2.**
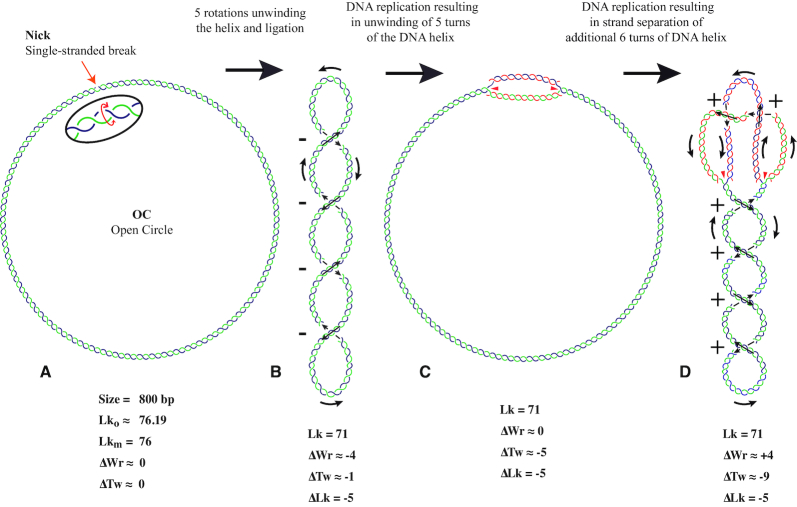
Concepts of ΔLk, ΔTw and ΔWr and interconversions between Wr and Tw in covalently closed DNA molecules. (**A** and **B**) When relaxed circular DNA is unwound by 5 turns at the site of the nick and then ligated the molecule becomes negatively supercoiled with ΔLk = −5. The torsional stress caused by unwinding is redistributed into changes of writhe (ΔWr) and twist (ΔTw). Where the Δ indicates the difference of Wr or Tw values, respectively, between torsionally relaxed form and the supercoiled form of otherwise identical DNA molecules. Due to a specific ratio between bending and torsional elasticity of DNA, ca 70–80% of ΔLk is converted into ΔWr. The rest of (ΔLk = −5) goes into decrease of DNA helicity (ΔTw ≈ −1). (**B** and **C**) In negatively supercoiled DNA, the strand separation during initiation of DNA replication is facilitated as it relaxes negative supercoils and thus decreases the elastic energy of the DNA molecules. In the shown case, upon strand separation of 5 turns of DNA helix the molecule reached the state with ΔWr = 0 and ΔTw = ΔLk i.e. the state that minimizes the elastic energy of circular DNA molecules. (**C** and **D**) When DNA replication continues in the absence of DNA topoisomerases, the DNA molecules become positively supercoiled and their elastic energy grows, which at some point will block a further progression of DNA replication. In the shown case, the strand separation extending over 6 additional turns of DNA helix resulted in the decrease of Tw by only 5 units as freshly replicated duplex portions form right-handed precatenanes permitting parental strands to twist once around the imaginary axis of the molecule. Therefore, the molecule reached the state with ΔWr = 4, ΔTw = −9 and ΔLk = −5. Notice that in the absence of DNA topoisomerases, the ΔLk stays constant.

Wr, on the other hand, is a measure of the winding of the axial trajectory of the DNA double-helix around itself (3). The Wr value of DNA molecules can be estimated by scoring self-crossings of the DNA axis. For this scoring, one needs to arbitrarily choose one of the two possible directions along the DNA molecule considered and then consistently analyze each perceived self-crossing for its sign (see Figure 1B). The resulting Wr is the sum of contributions of all self-crossings, where every positive and every negative self-crossing count as +1 and −1, respectively. The sign of the crossings stays the same when the chosen direction along the DNA molecule is changed to the opposite one, as at each crossing both direction arrows change their orientation and such changes keep the sign of the crossing constant. In contrast to the total score of inter-strand crossings that determines the Lk of covalently closed DNA and that stays the same irrespectively of the viewing direction, the total scores of self-crossings of the DNA axis change with the viewing direction. To account for this dependence on the viewing directions, the Wr is defined as the average scoring of self-crossing of a given configuration over all viewing directions that are equally redistributed in the 3D space. However, in some specific cases one direction of viewing is sufficient to get a good estimate of the writhe of supercoiled DNA molecules. This is the case when the molecules are adsorbed to a supporting thin film for electron microscopy analysis or to a mica surface for atomic force microscopy analysis (6,7). In these cases, molecules are flattened and then practically all directions of viewing result in the same number of perceived crossings. As it is conceptually simpler to discuss the situation where the sign and the number of perceived self-crossings of the DNA axis translate directly into writhe, the molecules presented in Figures 1 and 2 are meant to represent DNA molecules adsorbed to a flat surface, as it is the case of DNA molecules analyzed by AFM, for example. In this context, Figure 1C schematically presents a supercoiled DNA molecule that upon adsorption shows four negative intra-axial crossings and thus have its writhe nearly equal to −4.

Wr is a geometrical descriptor that characterizes a given momentary configuration of DNA molecules. Wr changes when DNA molecules change their shape as a result of thermal fluctuations, for example, or when helicases unwind a portion of covalently closed DNA molecules and DNA replication starts (see Figure 2). However, any change of writhe in covalently closed DNA molecules is always accompanied by the compensatory change of twist such that Lk, which is a sum of Tw and Wr, stays constant.

DNA molecules start writhing when their Lk is not equal to their twist. The writhing takes frequently the form of plectonemic supercoiling where the DNA double-helix helically winds around itself (see Figure 1C). When the Lk of a given DNA molecule is smaller than its Tw, the molecule becomes negatively supercoiled and its writhe has a negative sign. On the other hand, positively supercoiled DNA molecules, with positive writhe are observed when the Lk of a given DNA molecule is larger than its Tw. Superhelices in plectonemically supercoiled DNA molecules have a right-handed appearance for negatively supercoiled DNA and a left-handed appearance for positively supercoiled DNA molecules.

Within *Escherichia coli* cells, DNA is negatively supercoiled and forms right-handed plectonemes; however, roughly 50% of torsional stress resulting from the fact that DNA is actively maintained in the state where its Lk is smaller than its Tw, is constrained by DNA interaction with histone-like proteins and other proteins organizing bacterial chromosomes (8). Only the remaining half of that torsional stress is unconstrained and drives formation of right-handed plectonemes (9). In deproteinized covalently closed DNA molecules in solution, their torsional stress is absorbed in ∼20–30% by changes of DNA twist and in ∼70–80% by writhing, resulting in DNA supercoiling (10).

In Figure 1, we introduced the concepts of linking number, writhe and twist. While writhe measurements based on scoring of inter-duplex crossings can be performed by such techniques as TEM (transmission electron microscopy) or AFM (atomic force microscopy) (7,11–13), direct observations of DNA linking number and of DNA twist have been very difficult to achieve by these techniques. A notable exception consists of EM images of helical RecA–DNA complexes (14). However, the DNA twist in RecA–DNA complexes is not anymore of native DNA but of the DNA that is partially unwound and stretched by its interaction with RecA protein, resulting in a DNA helix with ∼18.6 pb/turn (15). More recently, though, the development of soft-touch atomic force microscopy permitted us to visualize directly DNA twist (16).

While TEM or AFM microscopy permit us now to measure DNA writhe and DNA twist of investigated DNA molecules, gel electrophoresis permits us to measure another characteristic of supercoiled DNA. This characteristic is ΔLk = Lk − Lk_o_, which is the difference between the actual Lk of a given DNA molecule and a hypothetical linking number (Lk_o_) that is equal to the intrinsic Tw of a given DNA molecule under given conditions or to Lk_m_, which is the closest integer value to Lk_o_ (see Figure 2).

ΔLk is a measure of the extent of DNA supercoiling of DNA molecules. If ΔLk of a given molecule is −5, for example, this indicates that this molecule is underwound by 5 turns as compared to its torsionally relaxed configuration (see Figure 2A). The main advantage of operating with ΔLk and not with the actual Lk of a given DNA molecule is that only the first one informs us about the level of DNA supercoiling. In addition, only ΔLk directly affects electrophoretic mobility of supercoiled DNA molecules and as such can be precisely determined using 2D gel electrophoresis (17). An additional advantage of using ΔLk is that frequently the actual Lk is unknown. If one is interested in the actual Lk of a given DNA species (e.g. of DNA molecules contained in a given topoisomer band in a gel) and knows its ΔLk, one needs to estimate its Lk_o_ and then calculate the Lk using the equation Lk = Lk_o_ + ΔLk. Lk_o_ is obtained by dividing the number of base pairs of the molecule by the number of base pairs it takes to make one complete DNA turn (3). It needs to be remembered though that the number of base pairs per turn is not constant but depends on external conditions such as temperature (18) and concentration of salts in the ambient solution (19). It also depends on the actual base-pair sequence of the DNA, where for example long polyA/polyT tracts have helical repeat of 10.1 ± 0.1 bp/turn and long polyG/polyC tracts have their helical repeat of 10.7 ± 0.1 bp/turn (20). However, under standard conditions used for biochemical experimentation DNA molecules with usual base composition, on average have ∼ 10.5 bp/turn (2,21).

Figure 2 shows an example of Lk_o_ and Lk_m_ calculation for a DNA minicircle with 800 bp. Assuming that this minicircle is composed of ‘average’ DNA with 10.5 bp/turn (20,21), we can estimate that the Lk_o_ of such a minicircle would be ∼76.19, which would be equal to the intrinsic Tw of that molecule. If such a relaxed DNA circle with a nick was ligated, it would have the highest chance to form a DNA topoisomer having an Lk = 76. The Lk of a DNA topoisomer that is closest to the torsionally relaxed state is known as Lk_m_ (2). The value of ΔLk characterizing DNA topoisomers usually indicates how much their linking number differs from Lk_m_. In such cases, all topoisomers have integer values of their ΔLk. This approach is followed in Figure 2. For some more detailed structural studies, however, especially when one has additional indications that Lk_o_ is determined with high accuracy, one can maintain the fractional ΔLk characterizing how the Lk of a given molecule differs from its Lk_o_. In such a case, the topoisomer of an 800 bp DNA minicircle with Lk = 76 would be described as having a ΔLk = −0.2 and the topoisomer with Lk = 77, for example, as having a ΔLk = 0.8.

Another standard measure of DNA supercoiling that is normalized for the size of the molecule is the ‘specific linking difference’ or ‘superhelical density’, denoted *σ* (2): *σ* = ΔLk / Lk_o_. Thus, for example a typical supercoiling density of purified bacterial plasmids is of about −0.05, which tells that DNA is unwound by about 1 turn per 20 turns of DNA helix (22).

An important source of DNA supercoiling in prokaryotic and eukaryotic chromosomes is transcription that induces axial rotation of DNA passing through transcribing RNA polymerases (23–26). Negative and positive supercoils are generated behind and ahead of advancing RNA polymerase, respectively (27). Moderate levels of negative supercoiling have a general transcription-stimulating role in prokaryotic and eukaryotic cells (28). A moderate level of positive supercoiling produced just ahead of transcribing polymerases has additional function in eukaryotic cells as it destabilizes nucleosomes present there, which facilitates RNA polymerase progression (29). In addition, DNA in bacterial chromosomes and bacterial plasmids are negatively supercoiled by the action of DNA gyrase (30). The topological state of prokaryotic genomic DNA is strictly regulated to maintain negative supercoiling (31).

### DNA topology during replication

Figure 2 shows that as strand separation starts at the origin of DNA replication and then progresses, the covalently closed negatively supercoiled DNA circle becomes first relaxed and then positively supercoiled. This is a direct consequence of the fact that in covalently closed DNA molecules the decrease of DNA twist, resulting from strand separation, has to be compensated by an increase of writhe according to the equation ΔTw = −ΔWr. At some point, a further size increase of the strand-separated region by one more base pair would require to increase the elastic energy of the replicated molecule by more than the energy that can be furnished by DNA helicases and DNA polymerases. At this point the replication would be blocked if the original Lk of the parental duplex were maintained unchanged. However, DNA topoisomerases can release the mechanical stress in replicated DNA molecules by permitting a progressive decrease of the original linking number between parental strands of replicating DNA molecules.

**Figure 3.**
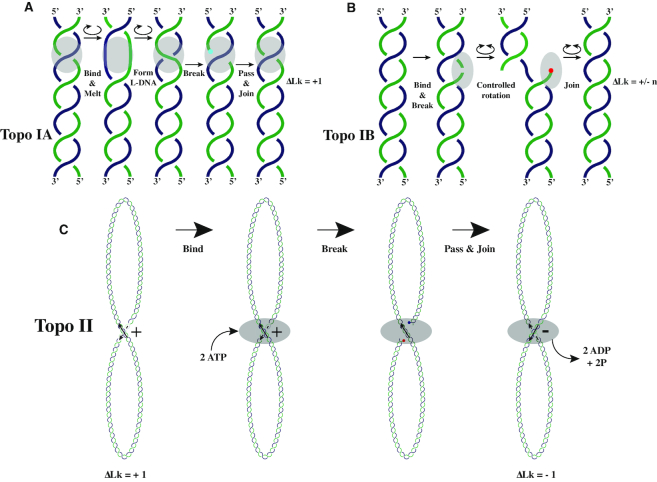
DNA topoisomerases. (**A**) Type IA DNA topoisomerases (topo IA) relax negatively supercoiled DNA regions that are excessively supercoiled and thus have their DNA helix destabilized. Binding of topo IA topoisomerases provokes further DNA destabilization and local strand separation. Separated strands, which are torsionally very flexible, start winding around each other in a left-handed way (L-DNA) as this partially releases the torsional stress in negatively supercoiled DNA. Topo IA introduces a transient cut into one strand in the destabilized region that winds in a left-handed way. The energy of opened phospho-diester bond is conserved by formation of a covalent bond involving 5′-end of the cut strand and one of tyrosines of topo IA. Subsequently, topo IA passes the intact strand of destabilized duplex through the opening in the cut strand. The continuity of strands is re-established by replacing the covalent bond connecting 5′-end of the cut strand with topo IA by a phospho-diester bond connecting it with 3′-end of the cut strand. The entire process results in the increase of the linking number by one, which decreases the excessive torsional stress in a negatively supercoiled DNA. (**B**) Type IB topoisomerases (topo IB) create transient swivels in DNA by cutting one strand and letting the DNA to swivel around the uncut strand (single chemical bonds can undergo unrestricted axial rotation). The region where DNA swiveling occurs is enclosed within the enzyme and since this enclosure limits the speed of torsional relaxation, the process is called controlled rotation. The energy of the cut phospho-diester bond is conserved by formation of a covalent bond involving 3′ end of the cut strand and one of tyrosines of topo IB. After a brief period of controlled rotation, the continuity of the cut strand is re-established by replacing the covalent bond connecting 3′-end of the cut strand with topo IB by a phospho-diester bond connecting it with 5′-end of the cut strand. The entire process results in the increase or decrease of the linking number by one or more units and can decrease torsional stress in negatively or positively supercoiled DNA regions. Panels (A) and (B) are based on Figure 1 in (32), where more details about mechanisms of DNA topoisomerases can be found. (**C**) Type II DNA topoisomerases transiently cut one duplex DNA region and move another duplex DNA region through the transient opening. Each passage changes the writhe by two units and this results in changing the linking number by two units. In principle, each passage can decrease or increase the linking number by two units. However, most of type II DNA topoisomerases preferably act on DNA crossings that have the geometry characteristic for intramolecular crossings with positive sign. Action of type II DNA topoisomerase at such crossings decreases the linking number of affected DNA molecules. The decrease of Lk is important for the relaxation of positive supercoiling generated during DNA replication and is also essential for the introduction of negative supercoiling in bacteria by DNA gyrase that is one of type II topoisomerases in bacteria.

**Figure 4.**
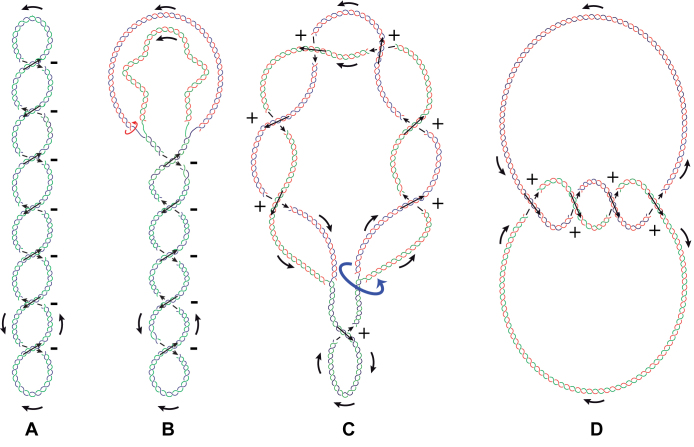
Changes of DNA topology during replication. (**A**) In most prokaryotes, native unreplicated molecules are negatively supercoiled. (**B**) Negative supercoiling facilitates the opening of the double-helix required for transcription and replication to begin and advance. This opening, however, generates positive torsional tension ahead of the fork. At the beginning, when unreplicated portions are sufficiently large, several molecules of DNA gyrase acting independently from each other can eliminate all positive torsional tension and even maintain negative supercoiling in the yet unreplicated portion of the molecules. Circular red arrow indictes that freshly replicated portions have the freedom of axial rotation due to the presence of single-stranded regions at replication forks.(**C**) As replication advances and there is less place for topoisomerases to act ahead of the replication fork, positive supercoiling accumulates in the unreplicated region. Rotations of the forks, indicated with a blue circular arrow, partially releases the torsional stress by formation of precatenanes that wind around each other in a right-handed way and have positive signs of their crossings as the direction of newly replicated portions of the molecules follows the direction of parental strands. (**D**) Once the replication is completed, fully replicated molecules form right-handed catenanes in which individual DNA circles are multiply catenated with each other. The sign of catenane crossings is positive as newly replicated molecules inherit the directions of parental strands. The unreplicated parental DNA double-helix is shown in blue and green while newly synthesized strands are depicted in red.

There are two general classes of DNA topoisomerases: Type I and Type II (see Figure 3A–C). Type I topoisomerases transiently cleave one of the strands of the DNA double-helix and before the cleaved strand is religated they either permit the other strand to pass once between the ends of the cleaved strand (Type IA) or permit controlled rotation of the DNA around the intact strand (Type IB) (32). Type II DNA topoisomerases transiently cleave both strands of the DNA double-helix and let another duplex DNA region from the same or other DNA molecule to pass between the ends of the cleaved duplex before its religation (see Figure 3C). As shown in Figure 4, the action of either type I or type II DNA topoisomerases on yet unreplicated portions of replicating DNA molecules permits the relaxation of torsional stress generated by strand separation. Therefore, in the presence of DNA topoisomerases the replication can proceed further, as the progressing strand separation is not opposed anymore by increasing torsional stress.

#### Formation of DNA catenanes

Since DNA replication is semi-conservative, each of two daughter double-stranded DNA molecules inherits one entire parental strand (33). How the parental DNA strands get unlinked during DNA replication is especially intriguing in the case of circular DNA molecules. Circular DNAs are popular in nature as bacterial chromosomes and plasmids are circular but also the DNA of chloroplasts and mitochondria are circular. In addition, the DNA of many viruses adopts a circular form during replication. Although in higher eukaryotes (such as humans), DNA molecules in individual chromosomes are linear, understanding of how living cells solve the topological problems connected to unlinking of DNA strands in circular DNA molecules helps us to understand how related topological problems arising during merging of freshly replicated DNA regions initiated at neighboring replication origins. In addition, even linear DNA molecules in eukaryotic chromosomes are organized into topologically constrained loops mediated by binding of specific proteins or interaction with membranes.

To discuss topological problems encountered during DNA replication let us first consider topological difficulties encountered during replication of circular DNA molecules and how these difficulties are solved in living cells. DNA replication necessitates progressive separation of initially paired strands. DNA helicases involved in DNA replication actively separate paired DNA strands using the energy gained from ATP hydrolysis (2,34). The process of strand separation is also driven by DNA polymerases that pair the separated strands with newly synthesized strands and by this prevent reannealing of the parental strands. In addition, in bacterial cells negative supercoiling facilitates the local strands separation that is needed for the initiation of DNA replication (3,35,36) (see Figure 4).

Since living cells contain DNA topoisomerases capable of relaxing excessive mechanical stress, such highly mechanically stressed molecules as schematically shown in Figure 2D are unlikely to appear*in vivo*. In addition, in bacterial cells the negative supercoiling facilitating strand separation can be maintained in replicating DNA molecules by several DNA gyrase molecules acting in parallel in the yet unreplicated portions of the DNA (Figure 4B). However, as DNA replication nears its completion the converging replication forks with associated DNA helicases and other protein factors progressively decrease the length of yet unreplicated portions, thus limiting the access of DNA topoisomerases to DNA located between the converging replication forks. This limits the risk that topoisomerase-introduced single or double-stranded breaks would not have enough time to be correctly religated before the passage of replication forks (37). If that would have happened, transient topoisomerase-mediated DNA breaks would be converted into long-lasting DNA breaks that are difficult to repair (38). With limited ability of DNA topoisomerases to act ahead of the replication forks the ongoing strand separation introduces torsional stress into replicating DNA molecules. This torsional tension can be released in part by swiveling of the forks allowing spreading of the torsional stress over larger portions of replicated DNA molecules behind the forks (39). Fork swiveling produces right-handed precatenanes in the replicated region where the inter-duplex crossings have a positive sign (Figure 4C). There is still a controversy as to whether fork swiveling occurs all the way during replication (40) or only at the end, as termination approaches (41). In any case, once the replication is completed, the newly synthesized sister chromatids form catenanes, which wind around each other in a right-handed way (Figure 4D). The right-handed winding of catenated rings, and also of precatenanes is a direct consequence of the fact that DNA forms a right-handed helix and that during replication of terminal portions of replicated DNA molecules the residual linking between two parental strands is converted into interlinking of freshly replicated portions where each contains one parental strand (42,43). The signs of crossings between catenated freshly replicated DNA molecules are positive as catenated rings keep the direction of parental strands and the crossings between parental strands in DNA are positive (see Figure 1). Type II DNA topoisomerases can decatenate these freshly replicated DNA molecules. This decatenation is necessary to allow the fully replicated DNA molecules to segregate into daughter cells.

#### How to get rid of positive supercoiling while keeping negative supercoiling?

In Figure 4, we show topological difficulties encountered during replication of small circular DNA molecules in bacterial cells. Nuclear DNA in eukaryotic cells, including infecting viral DNA, does not need to be constantly maintained in negatively supercoiled form, contrary to what is the case of bacterial DNA (44,45). In eukaryotic chromosomes a transient, local pulse of negative supercoiling facilitating transcription initiation can be generated when needed by the ejection of individual histone octamers in the vicinity of promoters of genes that need to be activated (46). Without the need to maintain a negative torsional tension in eukaryotic chromosomes, positive supercoiling arising during DNA replication in eukaryotic nuclei can be simply dissipated by topo IB-mediated free swivelling that removes any residual torsional stress in DNA. In bacteria, their DNA is actively maintained in negatively supercoiled form by the action of DNA gyrase and this negative supercoiling is essential for nearly all DNA transactions in bacteria (47–49). Therefore, a complete removal of torsional stress is not an option for bacterial DNA. In addition, action of topo IB topoisomerases in bacterial cells would be probably lethal as it would create a metabolic short circuit where many molecules of DNA gyrase would be using ATP to introduce negative supercoiling and topo IB would be continually relaxing this supercoiling. Not surprisingly, topo IB topoisomerases are absent in bacterial cells that have instead more sophisticated topo IA topoisomerases that work as torsional ratchets. Topo IA topoisomerases act on negatively supercoiled DNA that is too strongly supercoiled, which can happen behind transcribing RNA polymerases, for example (2,34). Too strong negative supercoiling, also known as hypernegative supercoiling, induces formation of R-loops where freshly synthesized RNA strand hinders transcription by prolonged pairing with its DNA template strand (50). Hypernegative supercoiling also provokes the formation of non-physiological alternative DNA structures such as cruciforms (51).

Binding of topo IA topoisomerase to too strongly negatively supercoiled DNA induces local melting of the bound region (52). Since a melted DNA region is torsionally more flexible than a base-paired region, the strands in the melted region flip to a left-handed configuration as such a flip is directed by a torque in negatively supercoiled DNA (52). The flipped region is then the site of action of topo IA topoisomerase, where one strand is cut and the other strand passes between the ends of the cut strand (see Figure 3). After the passage, the cut is resealed and as the result of this entire process, the linking number of the affected DNA molecule increases by 1 (53). This change of the Lk decreases the torsional tension in the molecules that are too strongly negatively supercoiled. Bacterial topo IA topoisomerases are unsuited though to act on positively supercoiled DNA. How then bacteria can prevent the accumulation of positive supercoiling generated by progressive strand separation during the process of DNA replication? To answer this question, we first need to present DNA gyrase that is a type II DNA topoisomerase specific of bacterial cells. This topoisomerase uses the energy of ATP hydrolysis to actively introduce negative supercoiling into DNA. Gyrase affinity to DNA induces right-handed wrapping of DNA around the C-terminal domain of GyrA subunit (54). In thus formed small DNA loop, incoming and outgoing DNA segments approach each other and form a positive crossing. Once that crossing is formed, gyrase transiently cleaves one of DNA duplexes forming the crossing and then passes the other duplex through the transient cut, before that cut is resealed. Each such reaction cycle decreases the linking number of the DNA by 2 and that is how negative supercoiling is introduced into bacterial DNA. In essence, DNA gyrase mechanism boils down to acting only on intramolecular positive crossings and converting them into negative crossings by the active passing of one double-stranded segment through the other, as schematically shown in Figure 3C.

In the absence of topological barriers blocking axial rotation of DNA, supercoils of opposite sign can simply cancel each other by twist diffusion. Therefore, each catalytic cycle of DNA gyrase can relax positive supercoiling resulting from the advancement of the replication fork by about 21 bp i.e. by two turns of DNA helix. In fact, one of main functions of gyrase is to relax positive supercoiling generated during DNA replication (30,39). However, many details of how this relaxation of positive supercoiling occurs are not established yet. Enzymatic studies of gyrase introducing negative supercoiling into DNA established that one reaction cycle decreasing the linking number of DNA by 2 takes about one second (55). On the other hand, bacterial replication forks advance with the speed of about 1000 bp per second, which translates into generation of about 100 positive supercoils per second ahead of each of the two replication forks in bacterial circular chromosomes. How then this slow gyrase action can relax positive supercoiling generated during DNA replication? A partial answer to this question is provided by the fact that gyrase is slow in a reaction where negative supercoils are introduced into relaxed but covalently closed DNA molecules. In that type of reaction, gyrase increases the elastic energy of DNA and therefore a significant work has to be done by the enzyme in that energetically uphill reaction. Of course, gyrase is able to do this work as it has at its disposal the energy gained from ATP hydrolysis. However, when gyrase acts on positively supercoiled DNA, such as this generated ahead of replication forks, the elastic energy of DNA molecules goes down and therefore this is energetically downhill reaction. Indeed, experiments testing the speed of DNA gyrase relaxation on positively supercoiled DNA showed that this speed is up to 10 times higher than the speed of introducing negative supercoils into relaxed but covalently closed DNA molecules (37). With that increased speed of DNA relaxation of positive supercoiling, individual gyrases are still ∼10 times too slow to relax positive supercoiling generated by individual replication forks. However, several gyrase copies can work ‘side-by-side’ leading to the additivity of their action. Several copies of gyrase could bind to the same positively supercoiled plectoneme and each could perform passages changing positive crossings into negative crossings. In fact, very recent studies using high resolution fluorescence aided imaging of gyrase in bacterial chromosomes revealed that about 10–12 copies of gyrase precede each replication fork (56). This recent finding explains how slow gyrases can completely offset the replication-generated positive supercoiling and thus allow the maintenance of negative supercoiling in the rest of a bacterial chromosome.

In the case when DNA gyrase activity would be not sufficient to completely eliminate the replication-generated positive supercoiling, that supercoiling is expected to promote formation of left-handed plectonemes. Left-handed plectonemes are preferentially recognized by another type II DNA topoisomerase that is present in bacterial cells and is known as topo IV (57). Topo IV action on left-handed plectonemes relaxes positive supercoiling. Therefore, topo IV can aid DNA gyrase in the relaxation of positive supercoiling generated ahead of the replication forks. However, the main function of topo IV is decatenation of freshly replicated DNA molecules (58), as it is presented later.

#### Topological domains

An additional mechanism that allows bacterial chromosomes to maintain their negative supercoiling despite ongoing DNA replication is the division of bacterial chromosomes into topological domains (59). Very early studies of bacterial chromosomes revealed that intentional DNA nicking relaxed topological stress in only a small portion of DNA around the nicking site (60). These earlier and more recent studies (59,61) showed that bacterial chromosomes are divided into topological domains, forming large plectonemic loops where specific proteins binding to loop bases could block axial rotation of the DNA. Each such loop is topologically independent from each other and can even maintain specific supercoiling density for a given loop, which is important for the regulation of expression of genes located in a given topological domain (62). Division of bacterial chromosomes into topological domains makes it that only these topological domains that are actually replicated need to deal with the strong injection of positive supercoiling. Since on average bacterial topological domains are 10 kb large (59) and bacterial replication forks advance with the speed of about 600 bp/s (63), therefore on average, replication forks spend about 20 s within a given topological domain. Therefore, supercoiling within individual topological domains is deregulated only during this short time and this would not be expected to significantly affect the cellular level of RNA and proteins encoded within a given domain. Once the DNA portion forming a given topological domain is replicated, the topological domains can reform again in each of the sister chromatids and can then regain their negative supercoiling thanks to the action of DNA gyrase.

#### How to decatenate right-handed catenanes without relaxing right-handed plectonemes?

As discussed above, in bacterial cells the relaxation of replication-induced positive supercoiling is achieved by ∼10–12 gyrase molecules acting ‘side-by-side’ ahead of moving replication forks (56). However, as two replication forks converge during replication termination, at some point there is no place for gyrase to bind and act ahead of the replication forks. Accumulating torsional stress can be then released by fork rotation resulting in the formation of precatenanes, which get converted into real catenanes upon termination of DNA replication (39). Formed catenanes need to be decatenated and it was shown that DNA topoisomerase IV (topo IV) is the main decatenase in bacteria (58). Topo IV is a type II DNA topoisomerase that makes transient double-stranded cuts and passes another duplex region, which finds itself near the cut site, through the transient cut. If topo IV would act on negatively supercoiled DNA and mediate passages between interwound duplexes, each passage would relax two negative supercoils and this would create a metabolic short circuit in a cell as gyrase would act to re-establish the original level of DNA supercoiling. One would have then topo IV using ATP to relax negative supercoiling and gyrase using ATP again to re-establish the negative supercoiling. As could be expected, a specific mechanism evolved that prevents topo IV from acting on negatively supercoiled DNA molecules. This mechanism is based on the recognition of the geometrical handedness of winding of DNA duplexes forming a superhelix. Topo IV DNA binding sites have such a geometry that topo IV can preferentially recognize and act on crossings in positively supercoiled DNA, which has a form of left-handed superhelix, whereas negatively supercoiled DNA, which forms right-handed superhelix, is a very poor substrate for topo IV action (57). As mentioned earlier, the preferential action on positively supercoiled DNA, forming left-handed superhelices makes that topo IV can partially substitute gyrase in its role of relaxation of positive supercoiling generated during DNA replication (37).

**Figure 5.**
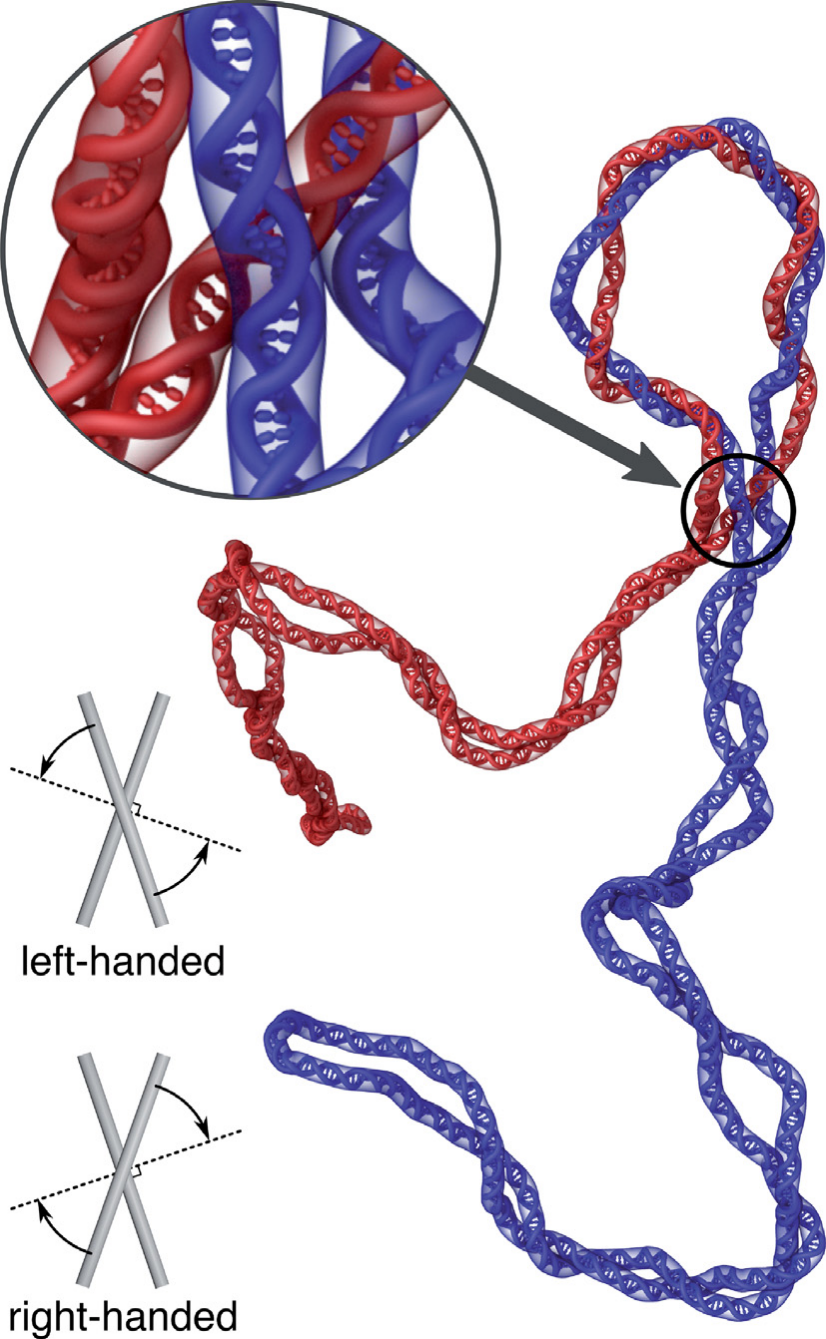
Simulation snapshot of negatively supercoiled postreplicative catenanes. The only left-handed crossing (indicated with an arrow) naturally forms at the place where topoisomerases preferentially acting at left-handed crossings can efficiently decatenate postreplicative catenanes. The insets schematically show crossings with left- and right-handed geometry. Left-handed crossings are these where one would need to turn the overlying segment counter-clockwise to make this segment perpendicular to the underlying segment. In right-handed crossings, the required rotation is in a clockwise direction. Of course, the required rotations can’t exceed 90°.

The evolutionary adaptation of topo IV to act on duplexes winding around each other in a left-handed way and avoiding duplexes winding around each other in a right-handed direction caused however a puzzle. How can topo IV decatenate freshly replicated DNA molecules that are winding around each other in a right-handed direction? An early proposal to solve this topo IV decatenation paradox assumed that right-handed winding of catenated DNA rings is very loose thus permitting formation of inter-ring crossings with left-handed geometry (64). Indeed, simulations of multiply-interlinked right-handed catenanes with loose winding of catenated DNA rings showed that in the presence of thermal fluctuation the catenated duplexes form relatively frequently inter-ring crossings with left-handed geometry. Therefore, topo IV acting on left-handed crossings could be still able to decatenate right-handed catenanes (64). However, that early proposal did not take into account that in bacteria both replicated DNA molecules forming right-handed catenanes are supercoiled. Later simulation studies (65), in which both DNA rings forming right-handed catenanes were negatively supercoiled, revealed that formation of plectonemes in individual rings sequesters loose DNA portions thus tightening the winding of catenated DNA rings (see Figure 5). This finding questioned the possibility that loose catenation of right-handed catenanes resolves the topo IV decatenation paradox. More recently, another simulation study investigated the handedness of all DNA–DNA crossings in right-handed catenanes formed by negatively supercoiled DNA molecules (66). That study revealed that even when right-handed catenane crossings are tight there is formation of intermolecular left-handed crossings in regions where supercoiled molecules enter into tight regions of their catenation (see Figure 5). Topo IV action at these left-handed crossings resolves then the topo IV decatenation paradox and explains how is it possible to decatenate right-handed catenanes without relaxing right-handed plectonemes (66).

### Replication fork regression and its reversal

**Figure 6.**
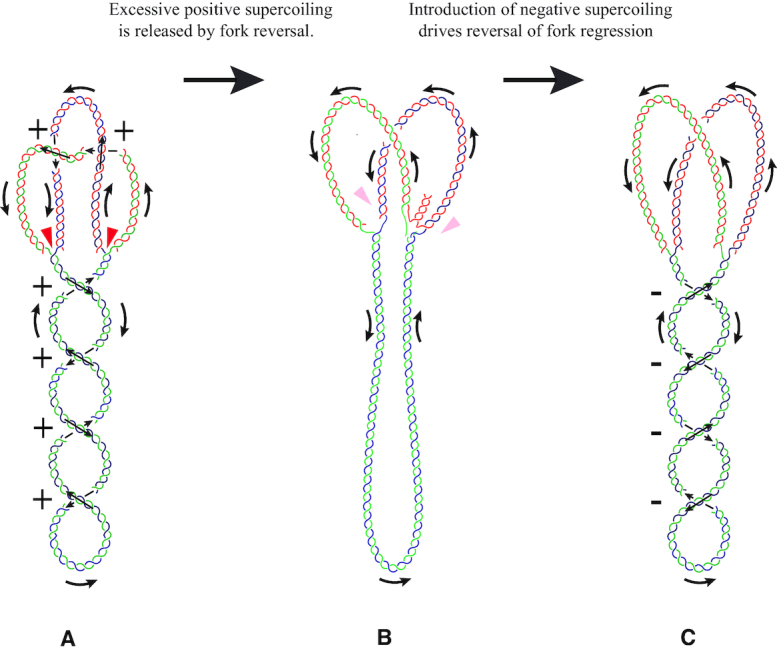
Replication fork regression and its reversal. (**A**) When the activities of cellular topoisomerases are perturbed, the positive supercoiling generated by ongoing DNA replication is not relaxed quickly enough. The resulting mechanical stress causes then the formation of positive supercoiling in the yet unreplicated portion of the DNA and of positive windings of the freshly replicated regions around each other. (**B**) Once the mechanical stress reaches a critical value, the replication is stopped and replisomes that normally prevent the parental strands from re-annealing are likely to be dislodged. At this point, mechanical stress resulting from positive supercoiling can be relaxed by the regression of one of the replication forks. (**C**) Action of DNA gyrase on DNA molecules with reversed forks causes the regression of fork reversal. In negatively supercoiled DNA molecules, it is energetically favorable to increase the region over which the parental DNA strands are separated and this can be achieved by the reversal of fork regression.

A very interesting example of the interplay between torsional stress and structural transitions within partially replicated DNA molecules is provided by the process of replication fork regression (67,68). As discussed earlier, the relaxation of positive supercoiling ahead of replication forks needs to be very efficient. If this relaxation is less efficient due to suboptimal conditions for action of DNA topoisomerases, for example, positive supercoiling can accumulate in unreplicated regions. Figure 6 schematically shows a partially replicated DNA molecule that accumulated positive torsional stress as the relaxation of positive supercoiling was not fast enough. The unreplicated portion of that molecule has the form of plectonemes with positive crossings and the replicated sister chromatids form precatenanes that crossings also have positive signs. As long as helicases present at both forks prevent the separated strands from reannealing these partially replicated DNA molecules can stay in that mechanically constrained configuration. However, if helicases at one or both forks would dissociate as a result of excessive mechanical stress, for example, such a molecule can decrease its elastic energy by the process of fork regression. The separated strands can reanneal and partially displace newly synthesized strands. Since the displaced, newly synthesized strands (colored red in Figure 6) are complementary to each other, they can pair. Therefore, with respect to base pairing the process is nearly isoenergetic as the number of formed base pairs is roughly the same before and after fork regression (the leading and lagging strands are usually of similar length). However, with respect to the elastic energy of the system, the regression is energetically favorable as it permits torsional relaxation of the unreplicated region and also permits bending relaxation of the sister chromatids that are not forced anymore to wind around each other. The fork regression is believed to have a physiological role and be triggered by excessive positive supercoiling (69,70). Regressed forks cannot support replication anymore and need to be reversed before the replication could continue. The reversal can be done by proteins involved in the process of DNA recombination (71). However, in the context of various effects of DNA supercoiling, the reversal can also be driven by re-establishment of negative supercoiling by the action of gyrase. When negative supercoiling is introduced into the unreplicated region of the molecule, that molecule would decrease its elastic energy by reversal of fork regression as this process effectively shortens the unreplicated duplex portion and thus decreases the deficit of parental-strand linking. During fork regression, the branch migration process leading to reversal of fork regression is essentially an isoenergetic reaction with respect to base-pairing as strands that separate in the extruded branch form new base pairs with parental strands. The reversal of fork regression is though energetically favorable when one considers elastic energy of the system as torsional and bending tension in negatively supercoiled replication intermediates is decreased. Once the fork is re-established it can bind all proteins needed to form an active replisome capable of sustaining DNA replication.

### Fully replicated catenanes

**Figure 7.**
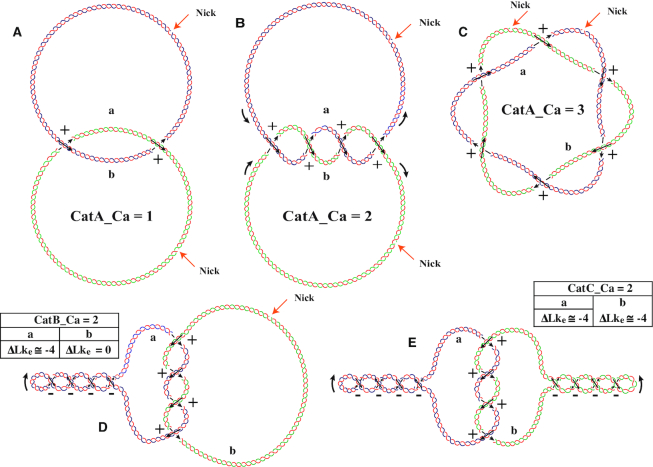
Different forms of DNA catenanes. (**A**) CatA catenanes composed of two fully replicated, torsionally relaxed rings that are singly interlinked and show two positive intermolecular crossings. (**B**) CatA catenanes composed of two torsionally relaxed rings that are interlinked twice and show four positive intermolecular crossings. (**C**) CatA catenanes composed of two fully replicated and torsionally relaxed rings that are interlinked three times with each other and show six positive intermolecular crossings. Notice that both rings wind around a common axis. (**D**) CatB catenane composed of one negatively supercoiled DNA molecule with four intramolecular crossings that is interlinked twice with torsionally relaxed DNA ring. Notice that the effective ΔLk (ΔLk_e_) of the left ring is approximately −4 while for the right ring, ΔLk_e_ = 0. (**E**) CatC catenane composed of two negatively supercoiled DNA rings that are interlinked twice with each other. Each ring shows four negative intramolecular crossings and contribute to four positive inter-molecular crossings. Notice that in this case, for both rings, ΔLk_e_ amounts to approximately −4. (a) and (b) denote the individual rings. The parental chains are in blue and green while newly synthesized chains are depicted in red.

Sundin and Varshavsky (42,43) were the first to observe that once replication is completed, the newly replicated circular DNA molecules are intertwined forming torus-type catenanes where the sister chromatids wind around each other showing positive crossings. Catenanes occur in three different forms (Figure 7): both intertwined circles could have single-stranded breaks or nicks (CatA), one circle could be nicked while the other is covalently closed (CatB) and both circles could be covalently closed (CatC). In addition, the circles could be intertwined just once (*Ca* = 1) or multiple times (*Ca* ≥ 2). *Ca* is the catenation number and it equals half of the signed sum of intermolecular crossings (2,42,43,65). TEM and simulation studies revealed that in multiply interlinked CatA catenanes their mutual windings tend to spread along the entire length of the molecules (43,72,73) (see Figure 7). However, in multiply interliked CatC catenanes supercoiling tends to confine all catenane crossings together (65,72–74) (see Figure 7).

Topo IV in prokaryotes and topo II in eukaryotes are responsible for the progressive decatenation of precatenanes and fully replicated catenanes (42,75–78). When these type II DNA topoisomerases are inhibited, molecules with high catenation numbers accumulate (65,75).

Interestingly, when two circular DNA molecules are multiply catenated, they acquire writhe that is induced by the catenation (79). Therefore, their equilibrium linking number, which is defined as the sum of the equilibrium twist and writhe of these molecules, is not equal to the equilibrium linking number Lk_0_ of identical but non-catenated DNA molecules. When catenated or knotted DNA molecules are supercoiled their extent of supercoiling is not expressed by ΔLk but by ΔLke (80,81). The ΔLke denotes the effective level of DNA supercoiling, which takes into account that multiply catenated DNA molecules begin to acquire their supercoiling starting from torsionally relaxed but significantly writhed configurations that these molecules attain when forming a given type of knot or catenane.

### DNA knots

**Figure 8.**
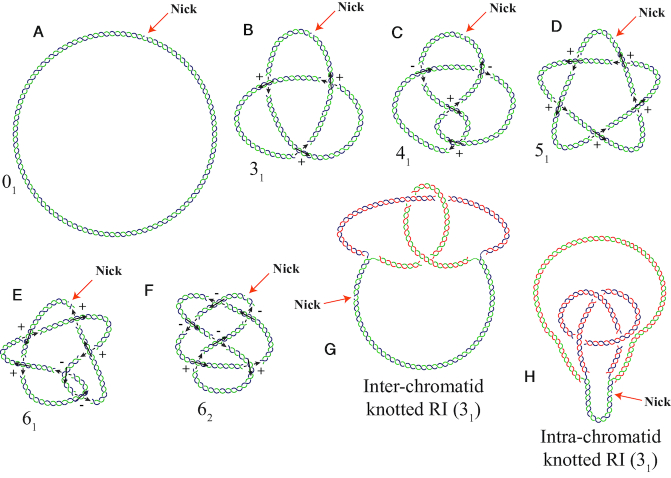
DNA knots and knotted bubbles. Cartoons representing nicked DNA rings forming various knots. (**A**) An unknotted molecule. (**B**) The classical trefoil knot with three positive nodes. (**C**) The so-called ‘figure of 8’ knot with two positive and two negative nodes that make this knot achiral. (**D**) A knotted molecule with five positive crossings. (**E**) A knotted molecule showing four positive and two negative nodes. (**F**) Another knotted molecule with six crossings but showing four negative and two positive nodes. Signs of the crossings are determined using the convention shown in Fig. 1B. (**G**) A partially replicated DNA molecule with a nick at the unreplicated region containing an inter-chromatid trefoil knot in the replicated region. (**H**) A partially replicated DNA molecule with a nick at the unreplicated region containing an intra-chromatid trefoil knot in the replicated region. The parental chains are in blue and green while newly synthesized chains are depicted in red. We used here Alexander-Briggs notation of knots (2). The notation is composed of two numbers. The first number indicates a minimal number of crossing a given knot can have and the second number written as subscript indicates the tabular position of a given knot among knots with a given number of crossings. Thus, for example, the notation 6_2_ indicates that the minimal number of crossings of this knot is 6 and its image can be found in topological tables of knots at the second position among knots with six crossings.

In everyday life, we are used to encounter knots on open strings such as on cables of ear buds. However, such entanglements are not knots in a strict topological sense, as with some patience such open-ended strings can be continuously deformed (without cutting and gluing) into a form without any entanglements. One can talk about knots in a strict topological sense when the knotted string has sealed ends as it is the case of circular single- or double-stranded DNA molecules and it is not possible anymore to continuously deform such a knotted string into a form without any entanglements. Interestingly, various types of knots have been observed on circular DNA molecules and Figure 8B–F schematically presents several types of DNA knots.

Liu *et al.* (82) were first who observed that treatment of single-stranded circular phage fd DNA *in vitro* with the *Escherichia coli* topoisomerase I (at the time called ω protein), yields a new molecular species that sediments faster than untreated DNA. Examination of this new species by electron microscopy showed that it consisted of single-stranded DNA rings containing at least three crossings. Liu *et al.* were also the first to observe naturally occurring double-stranded DNA knots in bacteriophage P2 DNA (83). Naturally occurring double-stranded DNA knots were also found in bacterial plasmids (84,85) and in circular minichromosomes of the budding yeast *Saccharomyces cerevisiae* (86). Thus, knots do form *in vivo* or can be created *in vitro* in both single- and double-stranded circular DNA molecules (2). The recognition of complex knots lies beyond the scope of this review (87). Some examples of simple knots are shown though in Figure 8. The topological convention of sign assignment of perceived crossings also applies to knots. Notice that orientation of the underlying and overlying direction arrows at each crossing are not independent from each other but result from assigning a consistent direction (see arrows) along the whole DNA molecule analyzed. In any case, the simplest type of knot that can be made in closed-circular DNA is a three-crossing knot or ‘trefoil knot’, so-called because when laid flat on a surface its structure has three lobes (Figure 8B). Agarose gel electrophoresis is probably the best method to resolve knotted molecules (88,89) and their electrophoretic behavior has been further analyzed using 2D agarose gel electrophoresis (17).

### Knotted replication intermediates

Winding of freshly replicated duplexes around each other in so called replication bubbles promotes formation of DNA knots (90). This was first observed in small bacterial plasmids containing two unidirectional origins facing each other, initiation of replication only occurs once per molecule. In some but not all plasmids, the replication fork initiated at the active origin stalls once it reaches the silent one. This phenomenon leads to the accumulation of partially replicated circular molecules containing an internal bubble (90). Digestion of these molecules with a restriction endonuclease that cuts outside the bubble in the unreplicated region leads to “linear" molecules containing an internal bubble. Analysis of these molecules by 2D agarose gel electrophoresis revealed that they form a ladder of bands suggesting that the replication bubble frequently forms different types of knots (90). This observation was confirmed by TEM (91). Replication knots could involve both sister duplexes (inter-chromatid knots) or only one (intra-chromatid knots). Similar results (Figure 8G and H) were obtained in bacterial plasmids of different sizes where the replication fork stalled at the TerE-TUS complex (69,92,93). Olavarrieta *et al.* also found that head-on collision of transcription and replication enhances the formation of these replication knots (94). It was later confirmed that topoisomerase IV, the bacterial decatenase (58,75), is responsible for making and resolving these replication knots (92,95). The formation of replication knots was recently analyzed using mathematical formalism of knotted theta-curves as partially replicated DNA molecules have the shape resembling Greek letter Θ (96).

### A competition between supercoiling and catenation or between supercoiling and knotting reveals itself as a beneficial cooperation

**Figure 9.**
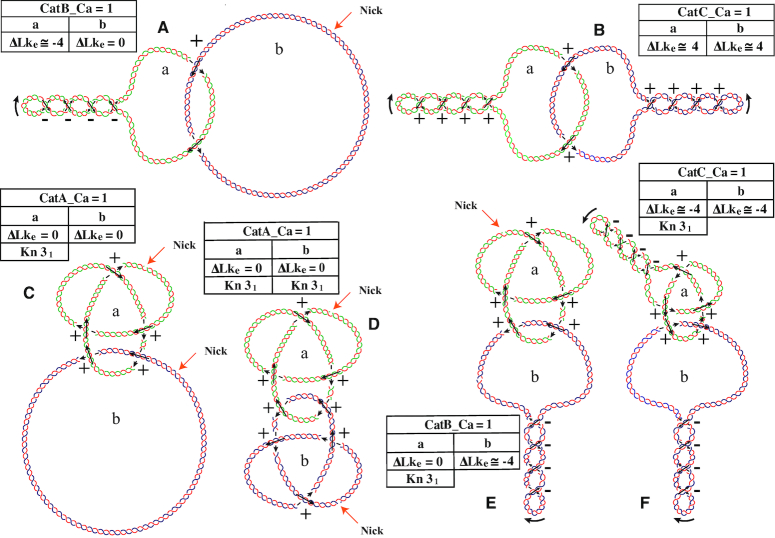
Supercoiled and knotted catenanes. (**A**) One fully replicated covalently closed and negatively supercoiled ring interlinked once with another fully replicated and relaxed ring. (**B**) Two fully replicated, covalently closed and positively supercoiled sister duplexes interlinked once. (**C**) A fully replicated and relaxed ring harboring a trefoil knot interlinked once with another fully replicated and relaxed ring. (**D**) Two fully replicated and relaxed sister duplexes both harboring a trefoil knot, interlinked once. (**E**) One fully replicated and relaxed ring harboring a trefoil knot interlinked once with another fully replicated covalently closed and negative supercoiled ring. (**F**) One fully replicated covalently closed and negative supercoiled ring harboring a trefoil knot interlinked once with another fully replicated covalently closed and negative supercoiled ring devoid of knots. (a) and (b) denote the individual rings. The parental chains are in blue and green while newly synthesized chains are depicted in red.

The interplay of supercoiling and catenation was investigated biochemically and by numerical simulations (65,79,97). The results obtained indicate that at least for small bacterial plasmids, the extent of catenation resulting from the intrinsic inability to unlink the DNA strands in the replication terminus, results in a high mechanical constraint that limits the ability of DNA gyrase to introduce supercoiling into catenated DNA circles. This large mechanical tension can be progressively released during topo IV-mediated decatenation of freshly replicated DNA molecules and therefore it contributes to driving the process of decatenation. However, as decatenation progresses the mechanical constraint due to interlinking decreases, this diminishes the driving force for decatenation but permits DNA gyrase to introduce increasing amounts of negative supercoils that re-establish the free energy gradient leading to decatenation (65). Curiously, in *Saccharomyces cerevisiae*, fully replicated catenanes were found to be transiently positively supercoiled (Figure 9B) during mitosis (76). Computer simulations showed that positive supercoiling of individual DNA circles in catenanes with right-handed crossings makes these catenanes more likely to undergo decatenation by type II DNA topoisomerases as compared to right-handed catenanes composed of negative supercoiled DNA circles (98).

The interplay of supercoiling and knotting, however, is harder to investigate using biochemical methods. This problem was analyzed in depth using numerical simulations (99,100). In short, it was found that tightening of DNA knots by supercoiling facilitates their recognition by type II DNA topoisomerases.

### Topological pinnacle: DNA molecules that are supercoiled, catenated and knotted at the same time

As previously mentioned, in the case of small bacterial plasmids when type II DNA topoisomerases are inhibited molecules with high catenation numbers accumulate (65,75). Surprisingly, 2D agarose gel electrophoresis revealed that in addition to the arc of CatAs, a second family of catenanes showing slightly faster mobility during the first dimension is systematically observed. Adams *et al.* suggested that this second family of catenanes were CatAs formed by one fully relaxed ring and another ring harboring a trefoil knot (75). Schematic drawing in Figure 9C represents this case whereas Figure 9F illustrates singly intertwined molecules that could be both supercoiled and knotted.

Indeed, the analysis of partially replicated molecules using biochemical methods and electron microscopy revealed that knotting occurs during replication that could involve a single duplex or both sister duplexes (75,90–92). Once replication is completed, inter-chromatid knots will convert into catenanes composed of two unknotted DNA rings, but intra-chromatid knots will result in catenanes formed by one unknotted ring and another ring harboring a knot (Figure 9C).

### Closing the loop

**Figure 10.**
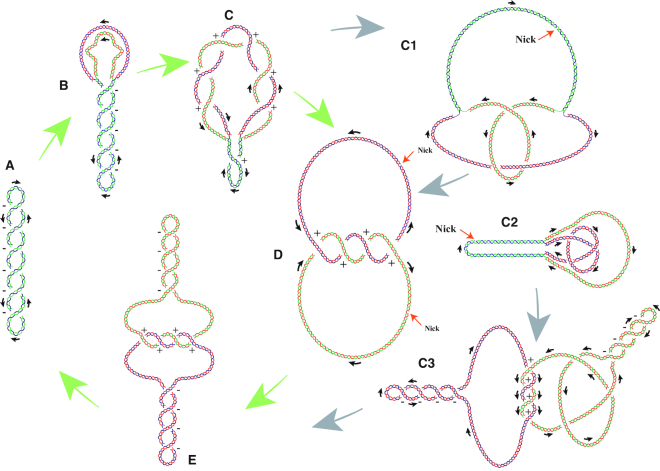
Topological transitions involved in closing the replication cycle of negatively supercoiled circular DNA molecules. The cycle marked with green arrows constitute a predominant ‘topology cursus’ taken by the majority of replicating circular DNA molecules such as bacterial plasmids. Gray arrows indicate facultative ‘topology cursi’ taken by part of replicating DNA molecules. (**A**) Not replicating, negatively supercoiled DNA molecule. (**B**) DNA molecule that initiated its replication. Negative supercoiling helps to initiate strand separation and continuous action of several DNA gyrase molecules acting within relatively large unreplicated portion of DNA molecule assures that the molecule can maintain its negative supercoiling, which favors further strand separation and relaxes positive supercoiling generated by strand separation. (**C**) DNA molecule toward the end of replicative strand separation. Unreplicated portion is too small to allow DNA gyrase(s) to bind and to relax positive supercoiling generated by replicative strand separation. Accumulating positive supercoiling induces formation of precatenanes that wind around each other in a right-handed sense and have positive signs of their crossings. (**D**) Freshly replicated DNA in which right-handed precatenane windings are converted to right-handed catenanes windings. (**E**) Once the continuity of freshly synthesized strands is achieved, each of catenated circles can acquire negative supercoiling due to action of DNA gyrase. In supercoiled catenanes, the windings between catenated rings are concentrated in one region that is exposed to topoisomerases mediating DNA decatenation. **C_1_** Partially replicated DNA molecule forming an inter-sister knot. After completion of replicative strand separation, the crossings resulting from entanglements of such a knot get converted into crossings between two catenated rings. **C_2_**. Partially replicated DNA molecule where a knot is formed within one freshly replicated sister chromatid. **C_3_**. Upon completion of replicative strand separation the molecules with one intra-chromatid knot get converted into catenated rings, where one ring inherits the intra-chromatid knot. When the knot is removed by action of DNA topoisomerases before the decatenation, this results in formation of supercoiled catenated rings shown in panel (E).

We discussed earlier the topological difficulties connected to DNA replication using as an example circular DNA, while devoting more space to bacterial systems where the role of negative supercoiling is better known. We discussed how these topological difficulties are resolved using specialized activities of various DNA topoisomerases. We discussed the effects of supercoiling, knotting and catenation on physical properties of DNA molecules during various stages of DNA replication. To provide a more global picture it is good to place all discussed events in the context of the entire replication cycle. Negative supercoiling is required for the initiation of DNA replication as it facilitates initial strand separation (see Figure 10A and B). Continuous action of DNA gyrase is needed to eliminate positive supercoiling generated by progressing replication forks. Negative supercoiling also facilitates decatenation of multiply catenated freshly replicated DNA molecules. According to our current understanding, DNA knots are not obligatory intermediates of DNA replication as they presumably form as the results of inadvertent DNA-topoisomerase passages occurring in crowded DNA molecules (91,96). However, once DNA knots are formed, DNA supercoiling helps to unknot them by shifting the equilibrium towards unknotting (80) and by localizing them and making them more likely to be recognized by DNA topoisomerases (101). Thus, despite the fact that DNA knots are deleterious and supercoiling-induced DNA crowding can be involved in the formation of some DNA knots (102), the overall effect of supercoiling is beneficial for knots avoidance (3,103).

## CONCLUDING REMARKS

Today, two-thirds of a century after Watson and Crick considered topological difficulties connected to replication of helical DNA molecules (1), we have a general understanding of how DNA topoisomerases make it possible that DNA replication proceeds without everything getting tangled. However, that understanding is in great part based on studies of bacterial plasmids, which due to their small size can be conveniently studied by gel electrophoresis, a technique that permits us to measure the extent of supercoiling, knotting and catenation generated during DNA replication (104). Our understanding of how entire bacterial chromosomes are replicated is more limited, as we still need to understand what drives the separation of freshly replicated chromosomes (105). What makes it that very floppy bacterial chromosomes after DNA replication attain essentially the same structure and position in progeny cells as they had in a mother cell so that even individual genes have their precise locations within bacterial nucleoid (106)? Our understanding of these questions progresses thanks to a rapid development of such methods as super-resolution optical microscopy (107,108) or chromosome conformation capture (61). Newly collected evidence points out that SMC (Structural Maintenance of Chromosomes) proteins such as bacterial MukBEF in association with type II DNA topoisomerases are deeply implicated in post-replicative chromosome segregation/folding and compartmentalization (109). The cooperation of type II DNA topoisomerases with eukaryotic SMC proteins, such as cohesin, in shaping interphase chromosomes and in the formation of TADs (topologically associating domains) was discovered only recently (110). In our opinion, the studies of cooperation between SMC proteins and topoisomerases will be the next important trend in DNA and chromatin topology (26).
